# Distinct and shared impacts of virulence plasmids on the phenotype and transcriptome in convergent carbapenem-resistant and hypervirulent *Klebsiella pneumoniae*

**DOI:** 10.1128/spectrum.04174-25

**Published:** 2026-06-09

**Authors:** Mingju Hao, Xiaodi Cui, Liya Feng, Ke Liu, Xiaohong Shi, Tengfei Long, Sarah E. Rowe, Yi-Tsung Lin, Liang Chen

**Affiliations:** 1 Department of Clinical Laboratory Medicine, The First Affiliated Hospital of Shandong First Medical University & Shandong Provincial Qianfoshan Hospital, Jinan, China; 2 Wen-shang County People’s Hospital, Wenshang, China; 3 Division of Clinical and Translational Therapeutics, School of Pharmacy and Pharmaceutical Sciences, University at Buffalo, Buffalo, New York, USA; 4 Department of Microbiology and Immunology, University of North Carolina, Chapel Hill, North Carolina, USA; 5 Division of Infectious Diseases, Department of Medicine, Taipei Veterans General Hospital, Taipei, Taiwan; 6 Institute of Emergency and Critical Care Medicine, National Yang Ming Chiao Tung University 34914, Taipei, Taiwan; Shenzhen University School of Medicine, Shenzhen, China

**Keywords:** hypervirulent *Klebsiella pneumoniae*, carbapenem resistance, virulence plasmid, CRISPR-Cas9, transcriptomic profiling

## Abstract

The global rise of convergent carbapenem-resistant and hypervirulent *Klebsiella pneumoniae* (CR-hvKp) represents a major clinical challenge, yet the role of virulence plasmids (pVirs) in shaping bacterial physiology and pathogenicity remains incompletely understood. Using a CRISPR-Cas9-based curing system, we precisely eliminated pVirs from two clinical CR-hvKp strains, sequence type 23 (ST23)-KL1 (*bla*_NDM-1_) and ST11-KL64 (*bla*_KPC-2_), with distinct genetic backgrounds. Loss of pVir conferred fitness advantages *in vitro*, reduced capsule production and hypermucoviscosity, and promoted biofilm formation, while markedly attenuating virulence in murine sepsis models. Despite this reduction, the pVir-cured ST23-KL1 strain retained higher virulence than the pVir-cured ST11-KL64 strain, underscoring the contribution of chromosomal background to pathogenic potential. Transcriptomic profiling revealed both shared and strain-specific transcriptional responses to pVir deletion, with broader perturbations observed in the ST11-KL64 strain. pVir removal had limited effects on antibiotic minimum inhibitory concentrations. Complementation experiments further demonstrated differential regulatory roles of the *rmpADC* and *rmpA2D2* operons in capsule expression and hypermucoviscosity across the two strains. Together, these findings establish pVirs as central determinants of CR-hvKp virulence and highlight complex host-plasmid interactions that influence bacterial adaptation and pathogenicity.

**IMPORTANCE:**

The emergence of carbapenem-resistant and hypervirulent *Klebsiella pneumoniae* (CR-hvKp) poses a critical threat to global health, yet the contribution of virulence plasmids (pVirs) to bacterial fitness and pathogenicity remains poorly defined. By employing a CRISPR-Cas9-based curing strategy, we dissected the role of pVirs in two genetically distinct CR-hvKp strains and uncovered their multifaceted impact on capsule production, hypermucoviscosity, biofilm formation, and virulence. Our findings reveal that pVir loss confers fitness advantages *in vitro* while attenuating virulence *in vivo*, with strain-specific transcriptional responses and differential regulation by *rmp* operons. These results underscore the complex interplay between plasmid-encoded and chromosomal determinants in shaping CR-hvKp pathogenicity and adaptation, offering mechanistic insights that may inform future therapeutic strategies targeting plasmid-mediated virulence.

## INTRODUCTION

Hypervirulent *Klebsiella pneumoniae* (hvKp) is a highly pathogenic microorganism with the ability to cause infections in both community and hospital settings (1, 2). First recognized as a cause of community-acquired pyogenic liver abscesses in Asia in the 1980s (3), it has now spread globally and causes a variety of infections, including liver abscess, bacteremia, pneumonia, and soft tissue infections (4, 5). Classic *K. pneumoniae* (cKp) strains, in contrast to hvKp, have long been recognized as opportunistic pathogens, primarily associated with hospital-acquired infections in immunocompromised patients. The cKp strains are often multidrug-resistant (MDR), with resistance to multiple antibiotic classes, including beta-lactams, fluoroquinolones, and aminoglycosides. The emergence of carbapenem-resistant *K. pneumoniae* (CRKP) in classic Kp strains has further exacerbated the challenge of treating these infections, making them a critical concern in healthcare settings worldwide (6).

Historically, hvKp strains were susceptible to most antibiotics, but now convergent multidrug-resistant and hypervirulent *K. pneumoniae* (MDR-hvKp), including those that are resistant to carbapenems, are emerging and posing serious health concerns (7). The emergence of MDR-hvKp mainly occurs via two major mechanisms: (i) acquisition of resistance genes by hvKp strains, such as the acquisition of a carbapenemase gene in the hvKp sequence type 23 (ST23)-KL1 clone; and (ii) acquisition of a virulence plasmid by classic multidrug-resistant strains, such as the KPC-producing ST11-KL64 clone (8–13).

The hypervirulent phenotype of hvKp has largely been attributed to the presence of a pK2044-like virulence plasmid (abbreviated as pVir in this study), which harbors the regulator of mucoid phenotype ADC genes (*rmpADC* and *rmpA2*), aerobactin (*iut*), and salmochelin (*iro*) siderophore biosynthetic genes and their cognate receptor genes (4, 14, 15). pVir-mediated hypercapsule formation, hypermucoviscosity (HMV), and hypersiderophore production are major virulence determinants of hvKp. In the hvKp K2 reference strain KPPR1, *rmpA* functions as an autoregulatory factor, activating the expression of downstream *rmpC* to stimulate capsule production, and *rmpD* drives the HMV phenotype (16–18). RmpD then binds to Wzc, a tyrosine kinase involved in capsule biosynthesis, which is essential for capsule polymerization and export. This interaction between RmpD and Wzc leads to the production of more consistent, longer polysaccharide chains, thereby leading to the HMV phenotype (18, 19). RmpA2, which shares approximately 80% identity with RmpA, plays a similar role to *rmpA* in enhancing capsule production (20, 21). In addition, a novel *rmpD2* gene was found downstream of *rmpA2* in most pVir plasmids, which contributes to HMV in the ST11-KL64 strain (22). The siderophore-associated genes include the aerobactin synthesis operon *iucABCD*, the salmochelin production gene cluster *iroBCDN*, and the outer membrane ferric aerobactin receptor gene *iutA* (23). Siderophores increase the ability of hvKp to acquire iron from the host (1), of which aerobactin is the dominant siderophore produced by hvKp (24). The pVir also harbors the gene *peg-344*, which encodes a putative transporter that is required for the full virulence of some hvKp strains in site-specific infection (2, 25). In addition, the pVir-borne tellurite resistance operon (*ter*) has been found to contribute to the fitness of gut colonization (26).

Despite being a hallmark of hvKp strains, the phenotypic and transcriptional impacts of pVir on different *K. pneumoniae* hosts remain unclear, partly due to the difficulty of generating isogenic pVir-cured strains in diverse clinical *K. pneumoniae* backgrounds. To address this gap, we employed a CRISPR-Cas9-mediated pCasCure plasmid-curing strategy to generate isogenic pVir-cured mutants. We then characterized the resulting phenotypic alterations and transcriptomic responses. This study establishes a systematic framework for dissecting pVir-associated functions across distinct *K. pneumoniae* lineages.

## RESULTS

### Bacterial strains

Two representative CR-hvKp isolates with distinct genetic backgrounds were selected from our collection of nearly 200 clinical *K. pneumoniae* isolates obtained from the First Affiliated Hospital of Shandong First Medical University between 2014 and 2019. JNQH373 is an ST23 KL1 hvKp strain carrying *bla*_NDM-1_, whereas JNQH97 is an ST11 KL64 CR-hvKp strain harboring *bla*_KPC-2_ (Table 1).

**TABLE 1:** Genotypic and mucoviscosity characteristics of the study strains, their pVir-cured derivatives, and complemented isolates

Strains	Genotype	String test^*b*^
JNQH97	ST11/KL64, O1ab	++
JNQH97PC^*c*^	pVir-cured JNQH97	−
JNQH97PC/rmpADC	JNQH97PC complemented with *rmpADC* loci cloned from wild-type JNQH97	−
JNQH97PC/rmpA2^*a*^D2	JNQH97PC complemented with rmpA2a*D2 loci cloned from wild-type JNQH97	−
JNQH97PC/rmpAD2	JNQH97PC complemented with *rmpA* and *rmpD2* ligation	++
JNQH373	ST23/KL1, O2a	+
JNQH373PC	pVir-cured JNQH373	−
JNQH373PC/rmpADC	JNQH373PC complemented with *rmpADC* loci cloned from wild-type JNQH373	++
JNQH373PC/rmpA2^*a*^ D2	JNQH373PC complemented with *rmpA2^a^D2* loci cloned from wild-type JNQH373	++
JNQH373PC/rmpAD2	JNQH373PC complemented with *rmpA* and *rmpD2* ligation	++

^*a*^ Corresponding genes were truncated.

^*b*^ For comparison of string test, we designated string length longer than 5 mm but shorter than 5 cm as +, longer than 5 cm as ++.

^*c*^ PC, virulence plasmid-cured strains.

### Genomic characteristics of the CR-hvKp strains

Genomic analysis showed that JNQH373 contains one circular chromosome and two plasmids of 55 kb and 280 kb, respectively. In contrast, JNQH97 harbors one chromosome and four plasmids, ranging from 83.3 to 217 kb. In JNQH373, the chromosomal *ybt1* (yersiniabactin siderophore biosynthesis) and *clb* (colibactin toxin biosynthesis) loci were located within ICEKp10. In contrast, JNQH97 harbored the *ybt3* allele in ICEKp3 on its chromosome. JNQH373 belonged to ST23 and carried the KL1 capsule and O2a O antigen, while JNQH97 belonged to ST11 and harbored the KL64 capsule type and O1ab O antigen. In JNQH97, the *ompK35* gene was truncated, and a di-amino acid insertion (glycine-aspartate) was found inserted in the extracellular loop 3 region of the OmpK36 protein. In contrast, both *ompK35* and *ompK36* were wild-type in JNQH373.

**Fig 1:**
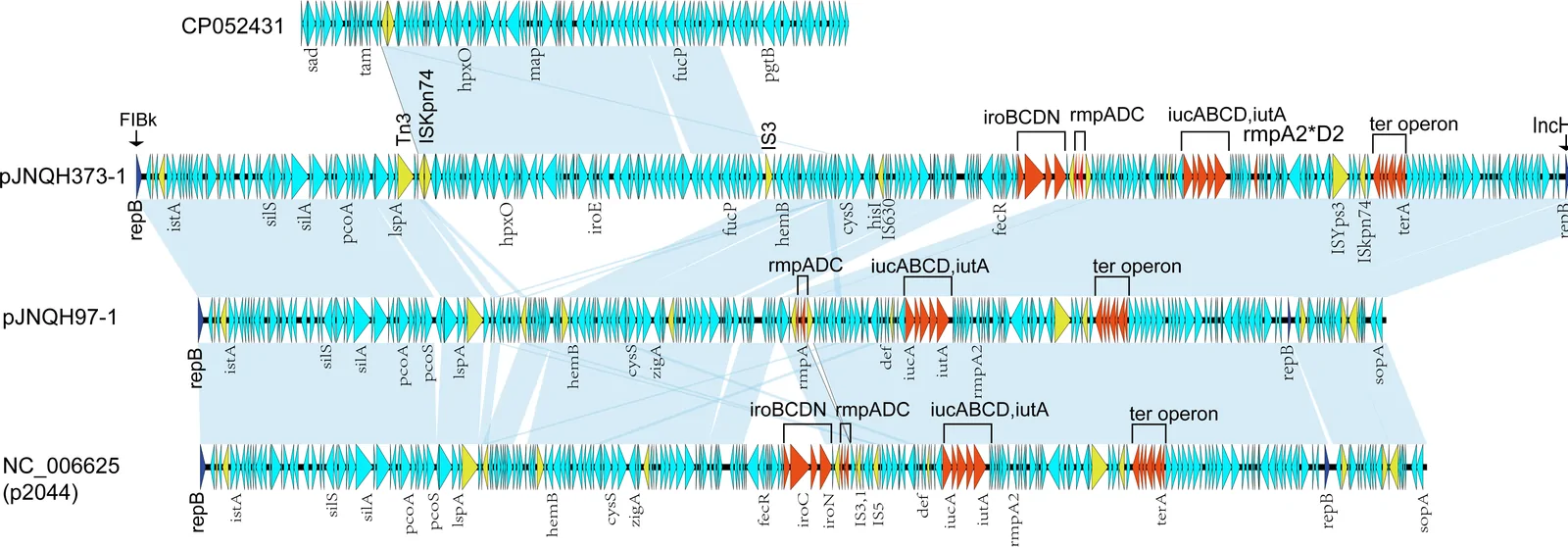
Structural comparison of the pVir-like plasmids from hvKp strains using complete plasmid sequences. *rmpA, rmpA2*, virulence-coding genes (*iro, iuc,* and *iut*), and *ter* operons were highlighted in red. Asterisks (*) indicate genes truncated due to premature stop codons. Shallow ocean shading indicates shared regions of homology. ORFs are represented by arrows and are colored based on predicted gene functions. Light blue arrows, plasmid scaffold regions; dark blue arrows, replication-associated genes; red arrows, virulence-coding genes; and yellow arrows, IS genes. The plasmid accession numbers are listed below the plasmid names.

JNQH373 carried the *bla*_NDM-1_ carbapenemase gene on an IncX3 plasmid (pJNQH373-2), while JNQH97 harbored the *bla*_CTX-M-65_*, bla*_KPC-2_*,* and *bla*_SHV-12_ genes on an IncR plasmid (pJNQH97-2). *bla*_LAP-2_, *qnrS1*, *sul2*, and *tet(A*) were found in another IncFII plasmid (pJNQH97-3) in JNQH97. Both strains contain pVir plasmids (pJNQH373-1 and pJNQH97-1) which had highly conserved plasmid synteny and structure (>99% nucleotide identity, 93% coverage) to the prototype virulence plasmid pK2044. The two pVir plasmids carry the IncHI1B and IncFIBk plasmid replicons, with sizes of 217,079 bp and 279,569 bp, respectively (Table 2). Both pVir plasmids carried the *rmpADC* and *rmpA2D2* loci; however, the *rmpA2* genes were truncated in both plasmids (Fig. 1). The aerobactin-encoding *iuc* and *iut* loci were present in both plasmids. However, the salmochelin-encoding *iro* gene cluster was absent in JNQH97. Additionally, a complete *ter* operon, which confers resistance to tellurite oxide (K₂TeO₃), was identified in both pVir plasmids.

**TABLE 2:** Genetic characteristics of the study strains displaying plasmid replicons, genome sizes, resistance, and virulence genes

Strain	Plasmid or chromosome	Total size	Inc type	Resistance and virulence genes
JNQH97	Chrome	5,465,676		*aadA2, bla*_SHV-11,_ *ybt3, ompK35^b^*
	pJNQH97-1^*a*^	217,079	IncHI1B/FIBk	*iucABCD, iutA, rmpADC, rmpA2^b^D2*
	pJNQH97-2	140,846	IncR_1/IncFII	*bla*_CTX-M-65_ *, bla*_KPC-2_ *, bla*_SHV-12_
	pJNQH97-3	83,385	IncFII	*bla*_LAP-2_*, qnrS1, sul2, tet(A*)
	pJNQH97-4	11,970	ColRNAI	
JNQH373	Chrome	5,359,407		*bla*_SHV-11_ *, ybt1, clb, ompK35, ompK36*
	pJNQH373-1^*a*^	279,569	IncHI1B/FIBk	*iroBCDN, iucABCD, iutA, rmpADC, rmpA2^b^D2*
	pJNQH373-2	55,296	IncX3_1	*bla*_NDM-1_ *, bla*_SHV-12_

^*a*^ Denote pVir-like plasmid.

^*b*^ Denotes these genes were truncated.

Further comparative genomics showed that these plasmids carried multiple insertion sequences, resulting in extensive rearrangement of pVir genetic elements. Interestingly, pJNQH373-1 contained a 62.1 kb region, flanked by IS*5* family transposase gene IS*Kpn74* and IS*3* family transposase IS*Ec36*, replacing a 10.9 kb fragment. BLASTn analysis indicated this region was nearly identical (100% coverage, 100% identity) to the *K. pneumoniae* chromosome of strain C16KP0122 (accession number CP052431). However, the fragment was not found in the chromosome of the host JNQH373 strain (Fig. 1).

### Impact of pVir curing on the *in vitro* fitness and virulence characteristics of CR-hvKp

**Fig 2:**
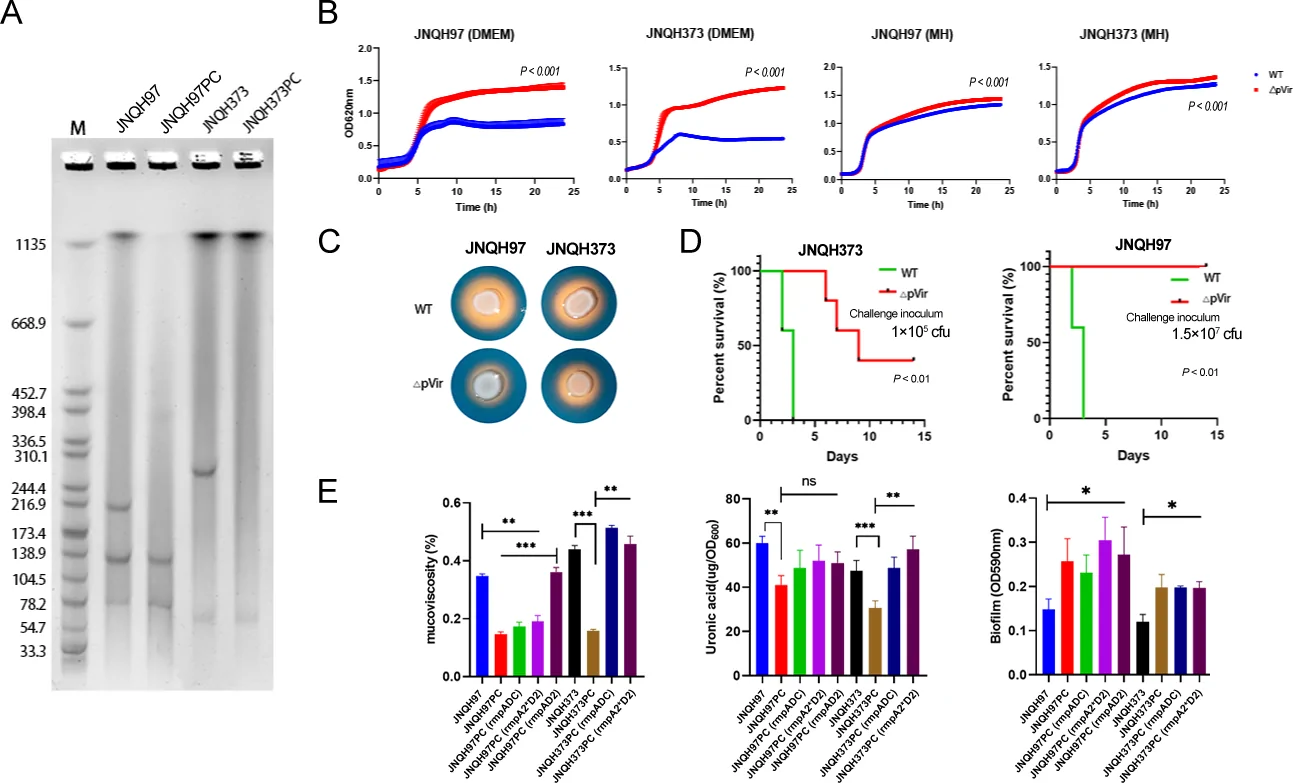
Phenotypic changes of hvKp strains after virulence plasmid curing and introduction of individual *rmp* locus. (**A**) S1-PFGE result of the pVir-cured and parental strains. “PC” denotes the pVir-cured strains. (**B**) Growth curve traces for wild-type and pVir-cured hvKp isolates. (**C**) Siderophore secretion experiment on CAS agar of hvKp isolates and their pVir-cured mutants. Light yellow areas around colonies indicate siderophore secretion. (**D**) Lethality assay of *Klebsiella pneumoniae* strains in a BALB/c murine infection model. The green line represents WT hvKp strains and the red lines represent their pVir-cured strains. The mice were challenged intraperitoneally with inocula of 1 × 10^5^ CFU for the paired WT/pVir-cured JNQH373, and 1.5 × 10^7^ CFU for the paired WT/pVir-cured JNQH97 strains. Each group consisted of five mice. The mortality of mice was observed over 14 days. (**E**) Mucoviscosity assay, uronic acid assay, and biofilm assay of hvKp strains carrying different virulence loci. Each data point was repeated three times (*n*  =  3). ns, not significant; *, *P* < 0.05; **, *P* < 0.01; ***, *P* < 0.001.

The pVir plasmids were successfully eliminated using our pCasCure system. S1-pulsed-field gel electrophoresis (PFGE) analysis confirmed complete plasmid loss, as evidenced by the absence of the pVir band in the cured derivatives (Fig. 2A). Growth-curve assays demonstrated that the parental strains harboring pVir exhibited significantly slower growth than their pVir-cured counterparts in both JNQH97 and JNQH373 (Fig. 2B, *P* < 0.001). Of note, growth assays showed that the difference was more pronounced in DMEM than in Mueller-Hinton (MH) broth, with the pVir-cured strains displaying a clearer growth advantage under this host-mimicking condition. However, neither the wild-type strains nor the pVir-cured derivatives were able to grow in M9 minimal medium under the conditions tested. These findings indicate that pVir imposes a significant fitness burden under *in vitro* conditions.

The string test revealed that parental hvKp strains formed a viscous string of >5 mm, whereas pVir-cured strains lost the HMV phenotype in both JNQH97 and JNQH373. These observations were consistent with the sedimentation assay results, where the pVir-cured mutants of JNQH97 and JNQH373 exhibited over a 50% reduction in mucoviscosity levels in comparison to their respective parental strains, as measured by optical density at 600 nm (OD_600_) (Fig. 2E).

Similarly, both pVir-cured strains displayed reduced siderophore production (Fig. 2C). Uronic acid quantification analysis revealed a ~ 35% reduction in uronic acid levels in both JNQH97 and JNQH373, indicating that pVir curing led to decreased capsule production (Fig. 2E). In contrast, biofilm production significantly increased in pVir-cured mutants compared to wild-type strains for both strains (Fig. 2E). Taken together, pVir curing resulted in reduced HMV, lower levels of CPS secretion, diminished siderophore production, and enhanced biofilm formation in both ST23-KL1 JNQH373 and ST11-KL64 JNQH97 strains.

**Fig 3:**
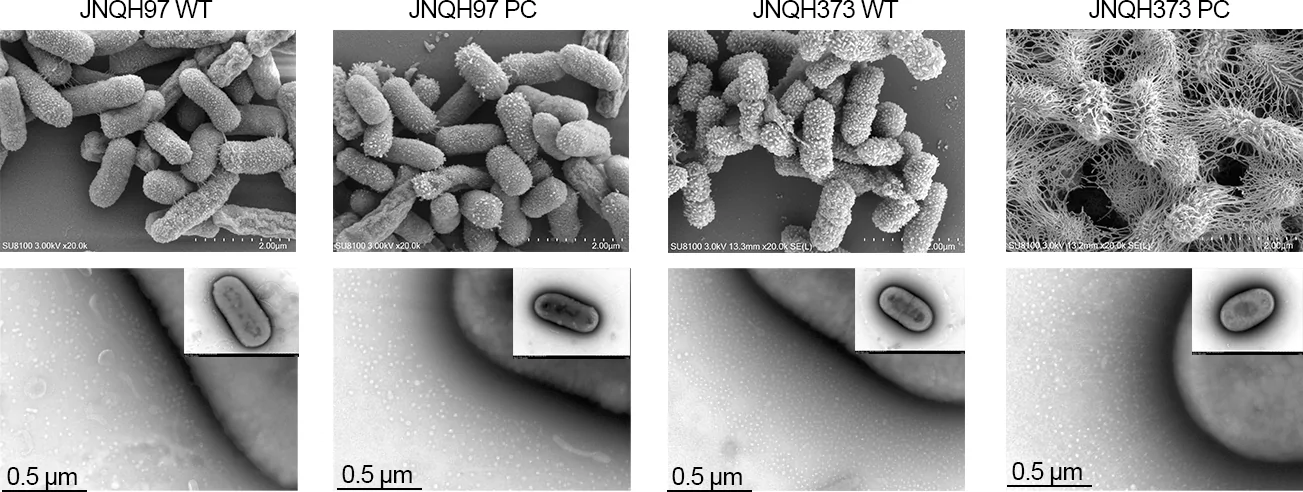
SEM images of wild-type and pVir-cured hvKp strains. SEM reveals that a thick variety of filamentous appendages in pVir-cured JNQH373. “PC” denotes the pVir-cured strains. TEM imaging of JNQH373PC showed surface filaments spread from the surface; however, they were absent in the parental JNQH373 strain. Both the wild-type and pVir-cured JNQH97 strains showed no discernible surface filaments. The scale bar is illustrated at the bottom of the images.

Scanning electron microscopy (SEM) analysis showed that the pVir-cured JNQH373 strain exhibited a pronounced hyper-piliated surface, whereas the wild-type parental strain lacked these prominent filaments (Fig. 3). The capsule-like material observed on the bacterial surface appeared diffuse in the pVir-cured strain, while remaining present but less apparent in the wild type. Transmission electron microscopy (TEM) imaging of pVir-cured JNQH373 showed surface filaments approximately 0.5 µm–2 µm in length, characteristic of typical type 3 fimbriae (27). However, the filaments were absent in the parental JNQH373 strain. In comparison, SEM and TEM analyses of both the wild-type and pVir-cured JNQH97 strains showed no discernible surface filaments, suggesting a fundamental difference in filamentous structure expression between JNQH373 and JNQH97, regardless of the presence or absence of the pVir plasmid.

The murine lethality assay revealed that the wild-type JNQH373 and JNQH97 caused 100% mortality by day 3. In contrast, pVir-cured JNQH97 did not cause any mortality over a 14 day period, and pVir-cured JNQH373 showed significantly reduced mortality (~60%, *P* < 0.01) compared to wild-type JNQH373 (Fig. 2D). However, the mortality rate of pVir-cured JNQH373 remained significantly higher compared to that of pVir-cured JNQH97, even when the latter was administered at a 150-fold higher dose.

### Distinct impact of rmpADC and rmpA2D2 operons on CPS and HMV in CR-hvKp strains

Co-transcription assays confirmed that *rmpADC* and *rmpA2D2* each operate as a single, polycistronic unit under the control of the promoter immediately upstream of *rmpA* or *rmpA2*, respectively. Curing pVir markedly reduced both HMV and CPS production in each background (Fig. 2E). In the ST23-KL1 (JNQH373) derivative, expression of either *rmpADC* or rmpA2*D2 fully restored mucoviscosity and CPS levels to those of the wild type. By contrast, in the ST11-KL64 (JNQH97) strain, individual complementation only partially rescued CPS synthesis and failed to recover HMV. Remarkably, introduction of the chimeric *rmpAD2* construct fully restored HMV in ST11-KL64. Lastly, all pVir-cured strains and those complemented with either *rmp* operon exhibited significantly higher biofilm formation compared with their parental counterparts (Fig. 2E).

### Impact of pVir curing on the antibiotic susceptibilities of CR-hvKp strains

Susceptibility testing of the pVir-cured and parental strains against several antibiotics was conducted, and the minimum inhibitory concentrations (MICs) are summarized in Table 3. Antibiotic susceptibility testing (AST) revealed that both parental hvKp strains were resistant to quinolones and most tested β-lactam antibiotics but remained susceptible to amikacin and tobramycin, with MIC values below 2 μg/mL and 1 μg/mL, respectively. JNQH97 was susceptible to ceftazidime/avibactam and aztreonam/avibactam, while JNQH373 showed resistance to ceftazidime/avibactam, consistent with the presence of *bla*_NDM-1_. Additionally, JNQH373 was resistant to tigecycline with an MIC of 4 μg/mL. However, no acquired or mutation-mediated tigecycline resistance genes were found. The pVir-cured mutants exhibited MICs similar to those of their parental strains.

**TABLE 3:** MIC profiles of the pVir-cured hvKp and their parental strains (μg/mL)^*a*^

Isolates	β-Lactams										Aminoglycosides		Quinolones		Others			
	TIM	TZP	CAZ	SCF	FEP	ATM	IMP	MEM	CAZ-AVI	ATM-AVI	AK	TOB	CIP	LEV	PMB	MH	TGC	SXT
JNQH97	>128	>128	>64	>64	>32	>64	>16	>16	4	4	<2	<1	>4	>8	2	>16	2	<20
JNQH97PC	>128	>128	>64	>64	>32	>64	>16	>16	4	4	<2	<1	>4	>8	2	>16	2	<20
JNQH373	>128	>128	>64	>64	>32	>64	>16	>16	>32	1	<2	<1	>4	4	1	>16	4	<20
JNQH373PC	>128	>128	>64	>64	>32	>64	>16	>16	>32	1	<2	<1	>4	4	1	>16	4	<20

^*a*^ TIM, ticarcillin-clavulanate; TZP, piperacillin tazobactam; CAZ, ceftazidime; SCF, cefoperazone-sulbactam; FEP, cefepime; ATM, aztreonam; IMP, imipenem; MEM, meropenem; CAZ-AVI, ceftazidime-avibactam; ATM-AVI, aztreonam-avibactam; AK, amikacin; TOB, tobramycin; CIP, ciprofloxacin; LEV, levofloxacin; PMB, polymyxin B; MH, minocycline; TGC, tigecycline; SXT, sulfamethoxazole-trimethoprim. All tests were performed in duplicate, and each test included three biological replicates.

**Fig 4:**
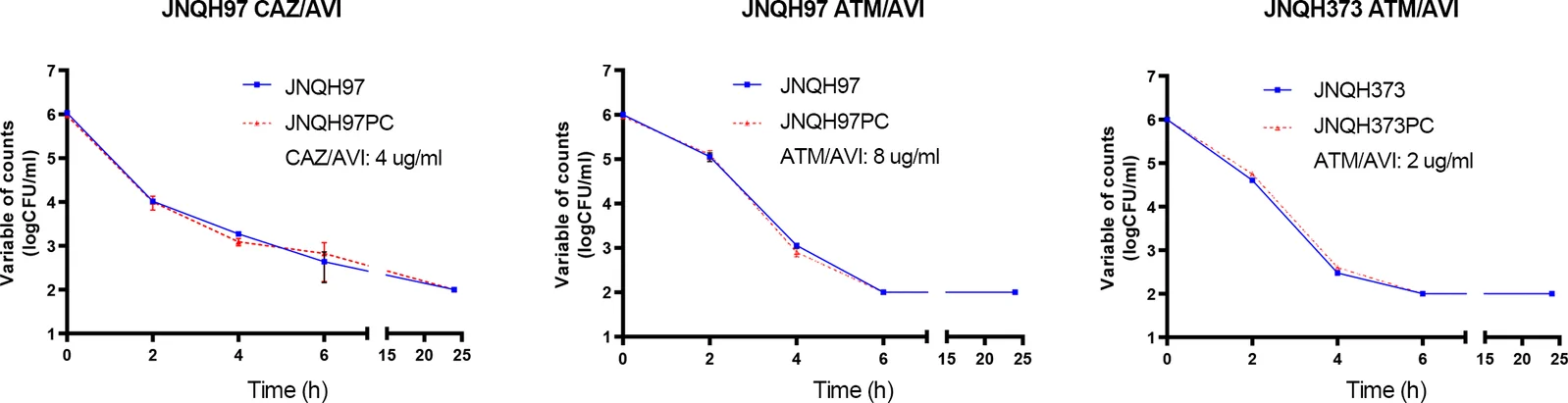
Time-kill curves of parental and pVir-cured hvKp strains exposed to CAZ/AVI and ATM/AVI. “PC” denotes the pVir-cured strains. CAZ/AVI and ATM/AVI showed similar bactericidal effects in both parental and pVir-cured strains. Antibiotic concentrations were indicated on each curve. Red dotted lines indicated pVir-cured strains, while solid blue lines indicated wild-type strains. Data points below the lower limit of detection (100 CFU/mL) were set to 2 log_10_ CFU/mL.

Time-kill curves showed that in both parental and pVir-cured JNQH97, ceftazidime/avibactam and aztreonam/avibactam achieved an approximately three- to four-log reduction in bacterial numbers within the first 6 h, which was sustained through 24 h. Similarly, aztreonam/avibactam exhibited comparable bacterial killing in both parental and pVir-cured JNQH373, with rapid killing in the first 6 h and sustained activity through 24 h (Fig. 4).

### Impact of pVir curing on genome-wide transcription changes

**Fig 5:**
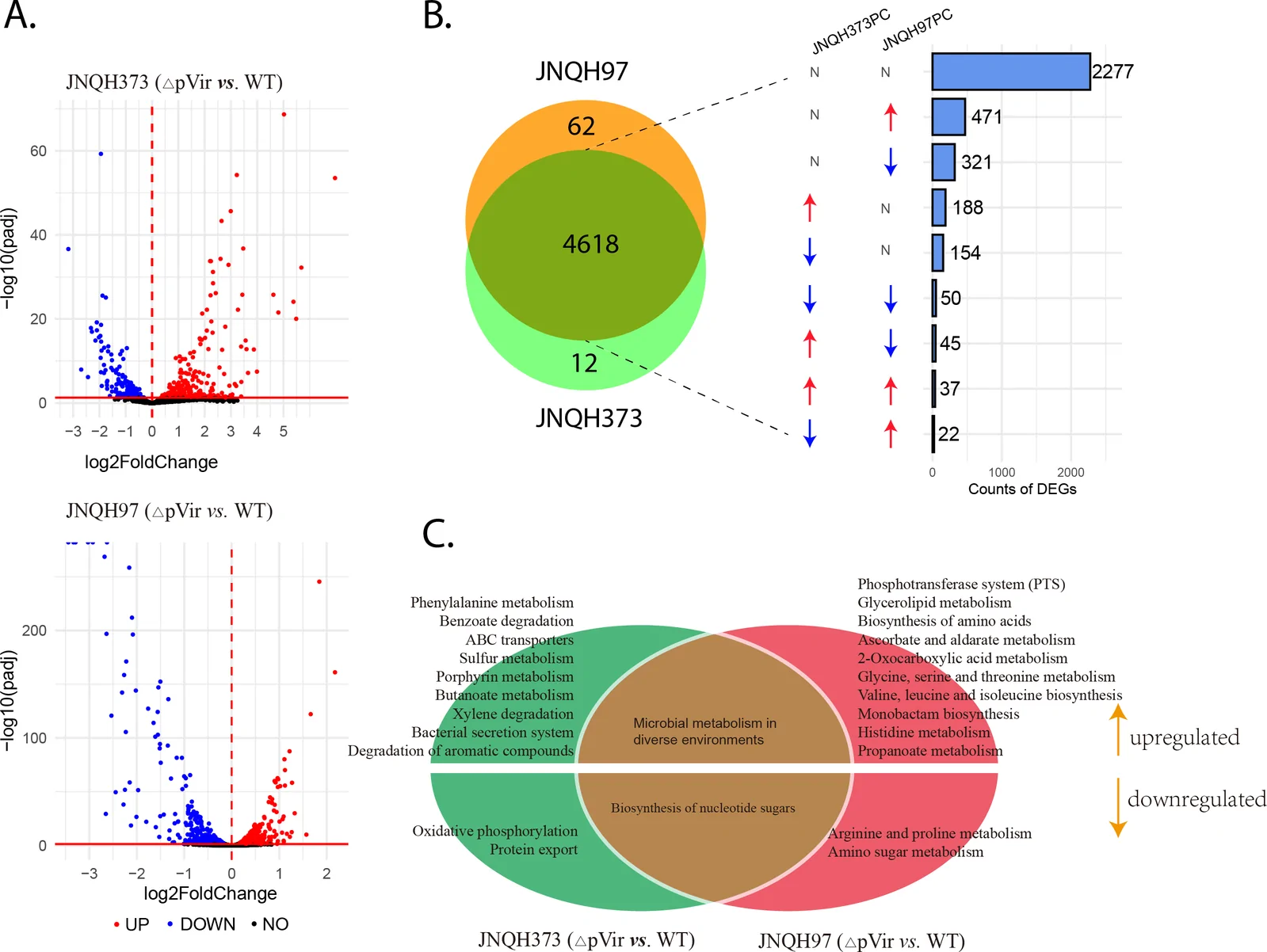
DEGs distribution and enriched metabolism pathways after pVir curing. (**A**) The volcano plot displays the differential gene expression results from RNA sequencing analysis following pVir curing in hvKp. Upregulated and downregulated genes are shown as red and blue points. (**B**) Orthologous clusters across JNQH97 and JNQH373 strains. Numbers of strain-specific and intersecting genes are illustrated. DEGs distribution of pVir-cured mutants relative to wild-type parent strains in JNQH373 and JNQH97 are shown. The numbers of DEGs are labeled on the right of the bar plot. (**C**) Kyoto Encyclopedia of Genes and Genomes (KEGG) pathway analysis revealed two pathways commonly affected in both strains following pVir curing. The left oval represents significantly enriched pathways in strain JNQH373, while the right oval represents those in strain JNQH97 following pVir plasmid curing. The overlapping region between the two ovals indicates shared differentially regulated pathways. Pathways located in the upper sections of the ovals are upregulated, whereas those in the lower sections are downregulated.

**Fig 6:**
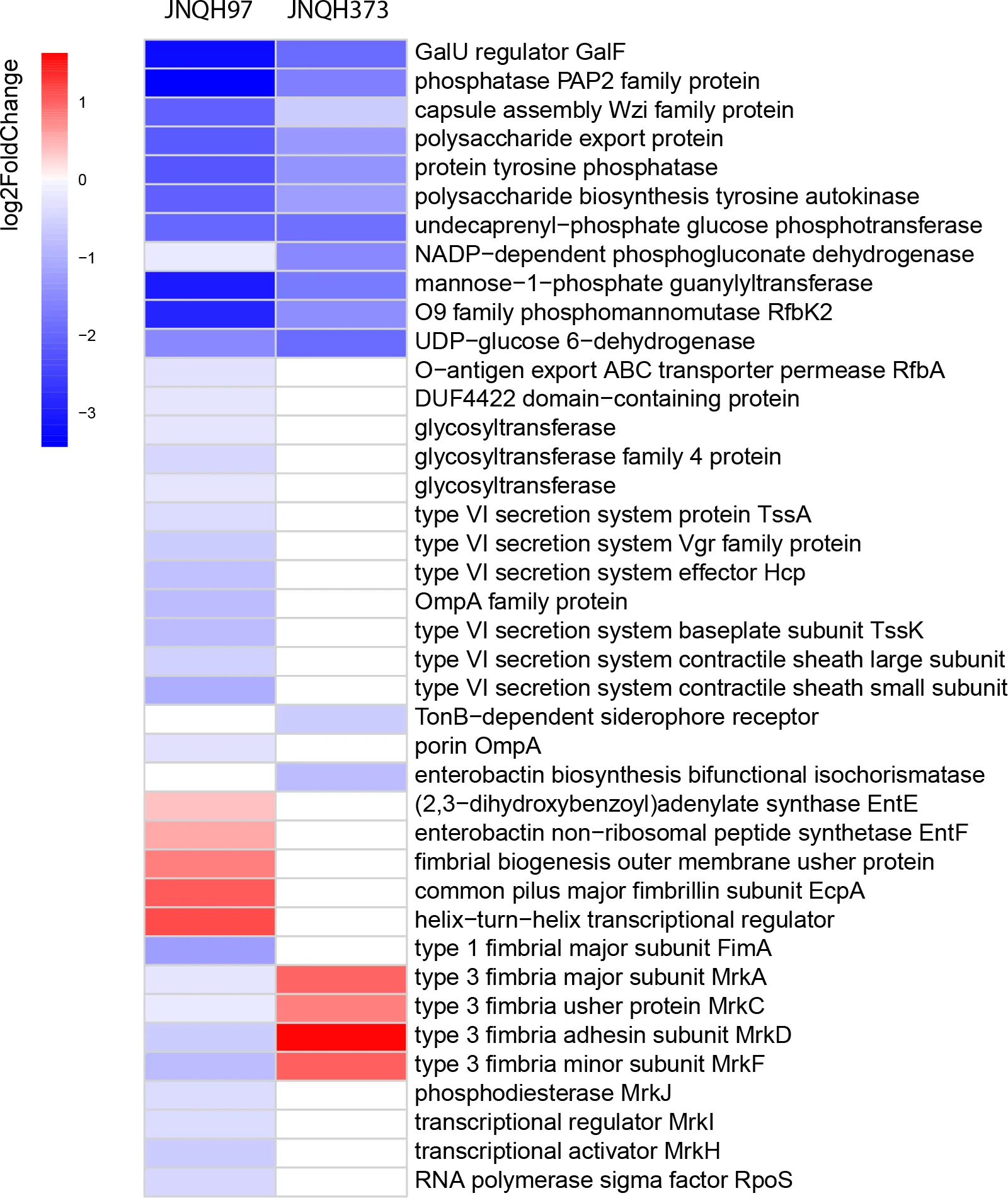
Expression alteration of virulence-associated genes after pVir curing. Virulence genes that did not significantly change were designated as white blocks.

To assess the transcriptional impact of pVir plasmid curing, RNA sequencing was performed on wild-type and pVir-cured strains of JNQH97 (ST11-KL64) and JNQH373 (ST23-KL1). In JNQH373, 549 differentially expressed genes (DEGs) were identified, comprising 297 upregulated and 252 downregulated genes (Fig. 5A). In contrast, JNQH97 exhibited a more extensive transcriptional response, with 1,182 DEGs—639 upregulated and 543 downregulated. Among the DEGs, 50 were consistently downregulated and 37 upregulated in both strains following pVir curing (Fig. 5B). The shared downregulated genes were primarily involved in CPS biosynthesis, including *galF, manC, wzi*, and genes related to polysaccharide export. These transcriptional changes are consistent with the observed reduction in CPS production and HMV in pVir-cured strains (Fig. 6). However, many DEGs were strain-specific: 471 upregulated and 321 downregulated genes in JNQH97 were not significantly altered in JNQH373, while 188 upregulated and 164 downregulated genes in JNQH373 were not altered in JNQH97. Moreover, 22 genes exhibited opposite expression trends between the two strains.

Distinct regulatory patterns were observed for genes related to adhesion and secretion systems. In JNQH373, type III fimbrial genes (*mrkA, mrkC, mrkD*, and *mrkF*) were significantly upregulated, correlating with enhanced biofilm formation and electron microscopy observations. However, these genes were downregulated in JNQH97, despite increased biofilm production (Fig. 6). Additionally, genes associated with the type VI secretion system were significantly downregulated in pVir-cured JNQH97 but remained unchanged in JNQH373 (Fig. 6).

KEGG pathway analysis revealed two pathways commonly affected in both strains following pVir curing (Fig. 5C). One was upregulated microbial metabolism in diverse environments, and the other was downregulated biosynthesis of nucleotide sugars.

In addition to these shared changes, strain-specific pathway alterations were identified (Fig. 5C). In JNQH373, pVir curing resulted in the upregulation of several metabolic pathways, including those involved in phenylalanine, sulfur, porphyrin, and butanoate metabolism, while pathways related to oxidative phosphorylation and the protein export system were downregulated. In contrast, in JNQH97, most of the affected pathways were related to amino acid metabolism. Specifically, histidine, glycine, serine, and threonine metabolism were upregulated, whereas arginine and proline metabolism were downregulated.

## DISCUSSION

The emergence of convergent CR-hvKp poses a significant threat to global public health. This study provides critical insights into the role of the virulence plasmid in shaping the phenotypic and transcriptional profiles of two distinct CR-hvKp strains, JNQH373 (ST23-KL1) and JNQH97 (ST11-KL64). JNQH373 represents a canonical hvKp lineage that has acquired a carbapenem resistance gene, whereas JNQH97 exemplifies an emerging convergent MDR lineage that has gained pVir. By employing a CRISPR-Cas9-mediated pCasCure system (28) to generate isogenic pVir-cured mutants, we elucidated the impact of pVir on bacterial fitness, virulence, and antimicrobial resistance, while also uncovering strain-specific regulatory mechanisms.

Our findings underscore the central role of pVir in maintaining the hypervirulent phenotype of the CR-hvKp strain, but impose a fitness cost to the host. The loss of pVir resulted in a significant reduction in HMV, capsule production, and siderophore activity in both JNQH373 and JNQH97, consistent with previous reports that pVir-encoded genes, such as *rmpADC*, *iucABCD*, and *iroBCDN*, are critical for the HMV phenotypes (14, 22). Notably, the pVir-cured strains exhibited reduced virulence in a murine sepsis model, exhibiting significantly lower mortality compared to their wild-type counterparts. However, the remaining virulence observed in the pVir-cured JNQH373 suggests that additional chromosomal factors, such as the KL and O antigen types, the yersiniabactin and colibactin loci, or different capsule types, contribute to its pathogenicity, particularly in the ST23-K1 lineage. In addition, the larger growth difference observed in DMEM than in MH broth likely reflects the greater physiological burden of pVir under nutrient-restricted conditions. In rich medium, this cost may be partially buffered by abundant nutrients, whereas in DMEM the metabolic demands associated with plasmid carriage are likely amplified, resulting in a more pronounced fitness disadvantage for the wild-type strains.

Our complementation experiments revealed distinct, lineage-specific regulatory roles of the *rmpADC* and rmpA2D2* operons in the production of CPS and HMV between ST23-K1 and ST11-KL64 strains. In the ST23-K1 strain JNQH373, either *rmpADC* or rmpA2D2* alone was sufficient to restore CPS and HMV levels, indicating functional redundancy between these operons. In contrast, in the ST11-KL64 strain JNQH97, neither *rmpADC* nor rmpA2D2* complementation could fully restore CPS or HMV, although *rmpADC* partially increased CPS production. Interestingly, in JNQH97, the *rmpA* gene appears capable of compensating for the truncated *rmpA2* gene, thereby contributing to the HMV phenotype. This compensatory effect may be due to the structural homology between RmpA and RmpA2 proteins (root mean square deviation [RMSD] 2.407 Å; Fig. S1). The inability of individual operons to fully restore CPS and HMV in this strain highlights the complexity of capsule regulation in convergent CR-hvKp lineages. These findings support the notion that the regulatory mechanisms governing CPS and HMV expression are lineage-dependent, consistent with recent studies (22, 29, 30).

The pVir plasmid did not substantially influence the antimicrobial susceptibility of strains JNQH373 and JNQH97, aligning with the well-established notion that classical hvKp strains typically retain broad susceptibility to most antibiotics (31, 32). Notably, time-kill assays demonstrated that ceftazidime/avibactam maintained similar bactericidal activity against both wild-type and pVir-cured JNQH97 strains, which harbor the *bla_KPC_* resistance gene. Likewise, aztreonam/avibactam showed equivalent efficacy against wild-type and pVir-cured JNQH373 strains carrying the *bla*_NDM_ gene. These results suggest that ceftazidime/avibactam and aztreonam/avibactam may serve as effective therapeutic options for treating infections caused by susceptible hypervirulent *K. pneumoniae* strains.

RNA sequencing revealed extensive transcriptional changes following pVir curing, with JNQH97 exhibiting a greater number of DEGs than JNQH373. This finding suggests that pVir imposes a more significant regulatory burden on the ST11-KL64 background, likely reflecting its more recent acquisition of pVir compared to the ST23-KL1 lineage. The downregulation of CPS biosynthesis genes in both strains was consistent with the observed decreases in capsule production and HMV. Nucleotide sugars are key precursors for the synthesis of bacterial capsular polysaccharides, lipopolysaccharides, and exopolysaccharides (33), which supports the observed reduction in capsule production. In contrast, genes associated with biofilm formation exhibited divergent expression patterns. In JNQH373, the type III fimbrial genes (*mrkA, mrkC, mrkD,* and *mrkF*) were markedly upregulated, whereas their expression remained unchanged in JNQH97. This discrepancy may reflect compensatory activation of alternative biofilm-associated pathways in JNQH97, including the upregulation of *ecpA* (34) and genes encoding chaperone-usher pathway proteins (35). Notably, genes linked to the type VI secretion system were markedly downregulated in JNQH97 following pVir curing, whereas their expression levels remained unchanged in JNQH373. These strain-specific transcriptional responses highlight the adaptability of *K. pneumoniae* to pVir loss and underscore the diverse regulatory mechanisms governing virulence and persistence.

The convergence of hypervirulence and multidrug resistance in *K. pneumoniae* represents a formidable challenge for clinical management (32, 36, 37). Our findings emphasize the need for targeted therapeutic strategies that address both virulence and resistance mechanisms. For instance, the development of inhibitors targeting pVir-encoded virulence factors, such as aerobactin biosynthesis or the *rmpA*-regulated capsule synthesis network, could attenuate hvKp pathogenicity without exerting selective pressure for resistance. Additionally, the strain-specific differences in CPS regulation warrant further investigation to identify lineage-specific vulnerabilities that could be exploited for treatment.

This study is limited by the use of only two representative CR-hvKp isolates, which restricts broad generalization. The other limitation of this study is that the transcriptomic analyses were mainly conducted in MH broth, a rich medium that may obscure phenotypes that are more evident under nutrient-limited conditions (38). Future studies incorporating additional strain backgrounds and nutrient-limited conditions will be essential to further elucidate the mechanisms underlying plasmid-mediated virulence, fitness, and antimicrobial resistance in hvKp.

Together, these findings provide a comprehensive view of pVir function in CR-hvKp and highlight the strain-specific regulatory complexity that shapes pathogenicity, underscoring the need for tailored strategies to mitigate the threat posed by this evolving pathogen.

## MATERIALS AND METHODS

### Case description

Strain JNQH97 was recovered from a sputum sample of a 57-year-old female patient who was admitted to the intensive care unit of a tertiary hospital in Eastern China in August 2014. She had a medical history of colon cancer, high blood pressure, and diabetes mellitus. Two days after admission, JNQH97 was isolated from a sputum sample, and 3 days after admission, she developed sepsis. During her 7 day hospitalization, she received multiple antimicrobial agents, including piperacillin-tazobactam and meropenem as empirical therapy. Despite treatment, her condition continued to deteriorate. The patient ultimately chose to discontinue medical care and was discharged against medical advice.

Strain JNQH373 was isolated from a blood culture of a 63-year-old male patient who had been admitted to a tertiary hospital in Eastern China in August 2019. The patient had a prior medical history of endovascular embolization for an arteriovenous malformation and evacuation of an intracranial hematoma. Upon admission, he presented with fever, respiratory distress, and dyspnea, necessitating orotracheal intubation and mechanical ventilation. Subsequently, a tracheostomy was performed, and closed thoracic drainage was administered. On hospital day 36, the patient experienced a sudden high fever, and blood cultures subsequently recovered a CRKP isolate, JNQH373. Antimicrobial therapy, including tigecycline and imipenem, was administered. However, the patient’s health condition continued to deteriorate. On hospital day 39, the patient was discharged at the request of his family for personal reasons.

### DNA sequencing and bioinformatics

The genomes of JNQH97 and JNQH373 were sequenced to closure using a combination of Illumina short-reads and Oxford Nanopore long-read sequencing. Genomic DNA was isolated using a Wizard HMW DNA Extraction Kit (Promega, Madison, WI, USA) and was subjected to Illumina NextSeq and Nanopore MinION sequencing. Hybrid *de novo* assembly was conducted using Unicycler v.0.49 (39). The whole-genome sequences were annotated by Prokka v.1.14.6 (40), followed by manual curation. Kleborate v.2.2 (41) was used for *Klebsiella* multilocus sequence typing, K locus, and O locus typing. The antibiotic resistance/plasmid replicon genes were identified using ABRicate v.1.0.1 (https://github.com/tseemann/abricate) using CARD (42) and PlasmidFinder (43) databases, respectively. Virulence loci (yersiniabactin, aerobactin, and other siderophore production systems) were identified using Kleborate v.2.2 in combination with the VFDB database (44). The complete sequences of virulence plasmids were compared with plasmid pK2044 (NC_006625.1), from K1 prototype strain NTUH-K2044 (45), using BLASTn and illustrated by Easyfig (46).

### CRISPR/Cas9-mediated pVir plasmid curing

To assess the phenotypic and transcriptomic impact of the pVir on the bacterial host, we used our CRISPR-Cas-mediated pCasCure system (28) to precisely eliminate the pVir from the two strains. CRISPR/Cas9-mediated pVir plasmid curing was conducted following our previously described method with minor modifications (28). Briefly, the 20 nt base-pairing region of an sgRNA targeting IncHI1B plasmid replicon was designed using the R package CRISPRseek (47). The sgRNA was introduced into the pCasCure backbone using PCR, restriction enzyme digestion, and ligation, as previously described (resulting in the pCasCure-N20_HI1B_ plasmid). The pCasCure-N20_HI1B_ was electroporated into the hvKp strain, followed by 1% arabinose treatment for 6 h at 37°C with shaking (200 rpm). The culture was plated on lysogeny broth (LB) agar containing apramycin. The complete curing of the pVir was verified by PCR screening using four primer sets targeting specific genes on the pVir plasmids, including *rmpA*, *iucA*, *iroN*, and the HI1B replicon gene (48). The pVir-cured strains were then streaked onto an LB agar plate containing 5% sucrose to remove pCasCure-N20_HI1B_. Lastly, next-generation sequencing confirmed the precise curing of pVir in both strains, without additional off-target mutations.

### S1-(PFGE)

To test the clearance of the pVir plasmid through the pCasCure system, we used S1-PFGE to confirm the successful curing of pVir. The parental and pVir-cured hvKp strains were digested with S1 nuclease after being embedded in 1% SeaKem Gold agarose gels, followed by plasmid DNA separation by PFGE as described previously (49). *Salmonella enterica* serotype Braenderup H9812 was used as a size marker. pVir-cured strains were also subjected to Illumina NextSeq sequencing as described above to check for potential mutations through plasmid curing.

### Co-transcription analysis

To assess whether the *rmpACD* and *rmpA2D2* operons were respectively co-transcribed as a single RNA, total RNA was isolated from strains JNQH97 and JNQH373 using the Bacteria RNA Isolation Kit (YALEPIC, China), following the manufacturer’s instructions. Subsequently, 200 ng of DNase-treated RNA was reverse transcribed into complementary DNA (cDNA) using the All-in-Mix 1st Strand cDNA Synthesis Kit (YALEPIC, China). Primer sets targeting the junctions of *rmp* operon components were used to perform PCR amplification on cDNA, genomic DNA (gDNA), and DNase-treated RNA controls. These reactions served to confirm both operon co-transcription and the absence of gDNA contamination. Promoter regions upstream of the *rmpA* and *rmpA2* genes were predicted using the BPROM tool (50).

### Cloning of plasmid-borne *rmp* homologs

The *rmpA*, *rmpA2* loci, and the upstream sequences were amplified with primer pairs rmpADC_locF/R and rmpA2D2_locF/R respectively, using plasmid DNA of strains JNQH373 and JNQH97 as the templates, respectively. These amplified fragments were ligated to the *pEASY*-T1 Cloning Vector (TransGen, China) by TA cloning and then transformed into *Escherichia coli* strain DH5α competent cells. The resulting plasmids recovered from the transformants were verified by Sanger sequencing and then transformed into pVir-cured JNQH97 and JNQH373, respectively. To examine the combined effect of *rmpA* and *rmpD2* in strain JNQH97, a chimeric *rmpAD2* construct was generated by PCR amplifying the two genes separately with primer pairs rmpADC_locF/rmpA_homo and rmpA2_homo/rmpA2_locR (Fig. S2). The two fragments were assembled by seamless cloning and then introduced into the pVir-cured recipient cells. The primers used for cloning are listed in Table 4.

**TABLE 4:** Spacer sequence and primers used for pCasCure editing, *rmp* cloning, and co-transcription analysis

Target	Sequence name	Primer sequence (5′−3′)	Product size (bp)
IncHI1B target			
*HI1B*	N20_HI1B_	TCAAAAACTCAACAGTAGAA	
Primers for validation of pVir curing			
*rmpA2*	rmpA2-F1	GGATGTGGCTTGACATTTCGGGGG	223
	rmpA2-R1	TTCATGGATGCCCTCCCTCCTG	
*HI1B*	HI1B-F1	TCGCTACTGCGATTGGGGGTCT	351
	HI1B-R1	GAAATGGGTGTGCTGGAGCCGT	
*iroN*	iroN-F1	CCGCAAAGAGACGAACCGCCTT	546
	iroN-R1	CGGGCAATCCCCGCTTTGACTT	
*iutA*	iutA-F1	AATCACCTGGGGGCTGGATGCT	683
	iutA-R1	CCGCACCTTCCACGCCGTAAAT	
Cloning of plasmid-borne rmp homologues			
*rmpADC* loci	rmpADC_locF	CCCCTCCCCACACATCTTTA	1,899
	rmpADC_locR	ACGCATTCATTCCCCTTCAT	
*rmpA2D2* loci	rmpA2D2_locF	TCTTAATCACACAGGGAAATGCT	
	rmpA2D2_locR	TGAGTCTGGTAGGTATGCATGT	
*rmpA-D2*	rmpA2_homo	GGGAGGGGATGTGAAGGAACTGCTTACTCTCCAGGAGGGA	
	rmpA_homo	TCCCTCCTGGAGAGTAAGCAGTTCCTTCACATCCCCTCCC	
Co-transcription assay			
*rmpAD* junction	rmpA-fw	GGGGGCGGTTTTATCCTAAAG	484
	rmpD-rev	GCGAGCGGAAGATTCTTTTTAT	
*rmpADC* junction	rmpC-rev	CGTTTGAATTAGGAGTGCATGC	863
*rmpA2D2* junction	rmpA2-fw	CGGATTGGAAATCATTACCCACAA	388
	rmpD2-rev	TGCGCCTCCTTCTTCTTGAT	

### Structural similarity assay

To evaluate structural similarity between the RmpA and RmpA2 proteins, 3D models were generated using AlphaFold (51) based on the wild-type amino acid sequences. Structural alignment of the predicted models was performed using PyMOL (52), and the RMSD value was calculated to quantify similarity between the aligned protein structures (53).

### Hypermucoviscosity assay

The mucoviscosity of the strains was determined using a string test and a sedimentation assay. A string test was performed by stretching the bacterial colonies on sheep blood agar plates using an inoculation loop. Strings of 5 mm or longer were defined as positive. For the sedimentation assay, cultures of *K. pneumoniae* were grown overnight in 5 mL LB medium at 37°C. The OD_600_ of cultures was measured, followed by spinning down at 2,500 g for 5 min. OD_600_ of the top 1 mL of supernatant was measured, and the sedimentation results were expressed as a ratio of the supernatant OD_600_ to that in the input culture.

### Growth curves

To evaluate the fitness impact of the pVir plasmid, bacterial growth was monitored in MH, the nutritionally restrictive DMEM broth, and M9 minimal culture, respectively. Overnight cultures were pelleted and washed twice with phosphate-buffered saline (PBS), then resuspended and adjusted to an initial OD_600_ of 0.005 in fresh medium. Aliquots of 200 μL from each prepared isolate were dispensed into individual wells of a sterile 96-well microtiter plate. Growth was tracked over a 24 h period at 37°C using a Multiskan FC Microplate Photometer (Thermo Scientific, IE), with OD_600_ readings recorded at regular intervals. Each strain was tested in triplicate per experiment, and the entire procedure was independently repeated three times to ensure reproducibility.

### AST

AST was performed using the automated VITEK 2 system (bioMérieux, Nürtingen, Germany) as described in our prior work (54). For ceftazidime-avibactam and aztreonam-avibactam, MICs were assessed via broth microdilution, with avibactam maintained at a fixed concentration of 4 μg/mL. MIC values were interpreted in accordance with the Clinical and Laboratory Standards Institute (CLSI) guidelines (M100-S32). Quality control was ensured using reference strains *E. coli* ATCC 25922 and *Pseudomonas aeruginosa* ATCC 27853. Each assay was conducted in triplicate across two independent days to confirm reproducibility. CLSI breakpoints were applied for all agents except tigecycline, for which the EUCAST epidemiological cutoff of >2 µg/mL was used.

### Biofilm formation assays

Biofilm formation was measured by crystal violet staining. In brief, overnight cultures were diluted 1:1,000 in LB medium, and a total volume of 200 μL was transferred to a vinyl “U”-bottom 96-well microtiter plates. Following incubation at 37°C for 24 h, the wells were washed four times with distilled water to rinse away the planktonic bacteria. The remaining adherent bacteria were stained with 125 μL of 0.1% crystal violet dye. After 10 min incubation, crystal violet was removed, and the wells were washed six times with distilled water. The stained biofilms were solubilized by 150 μL 30% glacial acetic acid in water, and the plate was incubated for 10 min at room temperature before the OD_590_ measurement with a microplate reader (Thermo Scientific, IE). At least three replicates were used for each sample, and the experiments were repeated twice.

### Siderophore secretion

Ability of strains to secrete siderophores was analyzed using the blue agar chrome azurol S assay (55). Briefly, overnight cultures were diluted 1:100 in 5 mL of fresh LB medium and grown to an OD_600_ of 0.6. Five microliters of culture was then inoculated on agar plates containing chrome azurol S-iron(III)-hexadecyltrimethylammonium bromide and incubated overnight at 37°C. Iron uptake was determined visually by color change from blue to yellow the following day.

### Capsule isolation and quantification

Overnight bacterial cultures (500 μL) were combined with 100 μL of 1% Zwittergent 3-14 (Aladdin) prepared in 100 mM citric acid buffer (pH 2.0). The mixture was incubated in a shaking water bath at 50°C and 170 rpm for 30 min. Following incubation, samples were centrifuged at 16,000 × *g* for 2 min. Three hundred microliters of supernatant was transferred to fresh 1.5 mL microcentrifuge tubes, and CPS was precipitated by adding 1.2 mL of absolute ethanol. Tubes were placed on ice for 30 min, then centrifuged at 16,100 × *g* for 10 min at 4°C. Pellets were air-dried for 30 min and resuspended in 100 μL of distilled water, followed by overnight incubation at room temperature.

Quantification of CPS was performed by measuring uronic acid content. For each sample, 20 μL of resuspended CPS was mixed with 120 μL of 12.5 mM sodium tetraborate (Aladdin) in concentrated sulfuric acid in PCR tubes. A standard curve was generated using serial dilutions of galacturonic acid (0 μg/mL–100 μg/mL, Aladdin). Tubes were heated at 100°C for 5 min with intermittent shaking, then cooled at room temperature for 15 min. Subsequently, 2 μL of 0.15% 3-phenylphenol (Aladdin) in 0.5% NaOH was added, and samples were allowed to equilibrate for another 15 min. After a final 5 min incubation with shaking, reactions were transferred to a 96-well microplate. Absorbance was recorded at 520 nm using a Multiskan FC Microplate Photometer (Thermo Scientific, IE). CPS concentrations were extrapolated from a glucuronic acid standard curve. All assays were performed in triplicate to ensure reproducibility.

### Time-kill experiments

We conducted time-kill assays to assess the kinetics of ceftazidime/avibactam (for the JNQH97 pair) and aztreonam/avibactam (for both strain pairs). Time-kill experiments were performed at 2 × MIC with CAZ/AVI (4 μg/mL), ATM/AVI (8 μg/mL for JNQH97, 2 μg/mL for JNQH373) for the susceptible strains. For CAZ/AVI and ATM/AVI testing, avibactam concentration was fixed at 4 mg/L. Precultures were prepared to achieve starting inoculum of 10^6^ CFU/mL in a total volume of 1.5 mL. The tubes were incubated on a shaker (200 rpm) at 37°C. Samples for viable counts were taken at 0 (before the addition of antibiotics), 2, 4, 6, and 24 h. Ten microliter samples were serially diluted as appropriate and plated onto Mueller-Hinton agar plates, which were placed in an incubator at 37°C for 24 h. Data points below the lower limit of detection (100 CFU/mL) were set to 2 log_10_ CFU/mL.

### *In vivo* murine lethality assay

To assess the impact of pVir on *in vivo* virulence, we used a murine lethality assay to evaluate the impact of pVir plasmid curing on the *in vivo* virulence of JNQH373 and JNQH97. Male BALB/c mice were challenged with 1 × 10⁵ CFU of JNQH373 or its pVir-cured mutant and 1.5 × 10⁷ CFU of JNQH97 or its pVir-cured mutant (25, 56) through intraperitoneal injection. The procedure for the murine lethality assay followed a previously described method (57). Male BALB/c mice aged 6 to 8 weeks were purchased from Beijing Vital River Laboratory Animal Technology Co., Ltd. (Beijing, China) and challenged intraperitoneally with a 1 mL sterile syringe. The first inoculation consisted of 1 × 10^5^ CFU in 100 μL of 1 × PBS for all wild-type or pVir-cured hvkp strains. Since no lethality was observed in the WT/pVir-cured strain JNQH97, the challenge inoculum was increased to 1.5 × 10^7^ CFU. The survival rate of the mice was observed daily for 14 days, and GraphPad Prism 8 was used to generate survival curves. The log-rank (Mantel-Cox) test was used for statistical analysis. Each experimental group consisted of five mice. To minimize pain and distress, mice were monitored at least twice daily following intraperitoneal bacterial inoculation and were humanely euthanized by cervical dislocation if they exhibited signs of severe illness or distress such as lethargy, ruffled fur, or labored breathing.

### RNA sequencing transcriptome analysis

RNA sequencing was conducted in pVir-cured and their parent strains to investigate the impact of pVir plasmid on hvKp bacterial host. Three replicates of each strain were performed. The bacterial strains were grown to mid-log phase at 37°C in LB broth. Cells were harvested and treated with RNAprotect (Qiagen), followed by extraction using UltraClean RNA isolation kits (MoBio). cDNA libraries were constructed with ScriptSeq Complete Gold kits (Epicentre Biosciences) and were sequenced on an Illumina HiSeq instrument. Raw reads from the sequenced libraries were subjected to quality control to trim the adaptor sequences using Trim Galore (version 0.4.5) and to filter out RNA sequences with SortMeRNA (58). The clean reads were mapped to the complete JNQH373 and JNQH97 genomes using the Rsubread package (59). The featureCounts function within the Rsubread package was used to summarize the data into gene-level read counts. Transcript abundance data were analyzed using the software package DESeq2 (60). The shrunken log fold changes were estimated using the method of approximate posterior estimation for generalized linear model (apeglm) (61). Differentially expressed genes were identified using an adjusted *P*-value (padj) threshold of <0.05. KEGG pathway annotation of differentially expressed genes was achieved by KOBAS-intelligence (62). Gene expression levels were subjected to Gene Set Enrichment Analysis using clusterProfiler 4.0 (63). Comparative analysis of orthologous clusters across JNQH97 and JNQH373 strains was determined by OrthoVenn2 web server (64).

### Scanning and transmission electron microscopy

For scanning microscopy, overnight cultures of bacteria were harvested at 2,000 × *g* for 7 min and washed with 1 × PBS (pH 7.3). The bacteria were then fixed in 2.5% glutaraldehyde (Sigma-Aldrich) in 1 × PBS (pH 7.3) for 2 h at 4°C. Then the samples were rinsed in 1 × PBS and subsequently post-fixed in a solution of osmium tetroxide at 4°C. After washing in distilled water, the samples were dehydrated in a series of ethanol solutions of ascending concentrations up to 100% and dried at the critical point of liquid CO_2_. Lastly, the samples were mounted on aluminum specimen stubs using carbon adhesive tape, sputter-coated with a 15 nm gold/palladium layer, and examined using scanning electron microscopy. For transmission electron microscopy, the bacteria were deposited onto a copper grid coated with a carbon film and allowed to adhere for 3 to 5 min. Subsequently, they were subjected to staining using a 2% phosphotungstic acid solution for 1 to 2 min. Following this, the copper grids were air-dried at room temperature before being examined using transmission electron microscopy.

### Statistical analysis

An unpaired two-sided Student’s *t*-test was performed to analyze the statistical difference between the levels of viscosity, biofilm formation, and CPS production. To compare the growth curves between the wild-type and pVir-cured hvKp strains, nonlinear regression analysis was performed using a logistic growth model. The difference in BALB/c murine survival between groups was assessed using the log-rank (Mantel-Cox) test, which evaluates statistical significance across Kaplan-Meier survival curves. A threshold of *P* < 0.05 was considered statistically significant. The statistical analysis was performed with GraphPad Prism 8.

## Data Availability

The genome sequences of hvKp strains JNQH97 and JNQH373, along with the RNA-Seq data for the pVir-cured mutants and their respective wild-type parental strains, have been deposited in Figshare. For strain JNQH97, raw Illumina RNA-Seq data are labeled as follows: Wild-type: J97_1, J97_2, J97_3; pVir-cured mutant: J97_4, J97_5, J97_6. For strain JNQH373, raw Illumina RNA-Seq data are labeled as: Wild-type: W373_1, W373_2, W373_3; pVir-cured mutant: M373_1, M373_2, M373_3. Each condition includes three biological replicates. The data set is available at https://doi.org/10.6084/m9.figshare.30446609).

## SUPPLEMENTAL MATERIAL

Figure S1 spectrum.04174-25-s0001.tif 3D structure and similarity alignment of rmpA and rmpA2 protein.

Figure S2 spectrum.04174-25-s0002.tif Cloning strategy for rmpADC, rmpA2*D2, and rmpAD2 operons.

Supplemental legends spectrum.04174-25-s0003.docx Descriptive legends for Fig. S1 and S2.

ASM does not own the copyrights to Supplemental Material that may be linked to, or accessed through, an article. The authors have granted ASM a non-exclusive, world-wide license to publish the Supplemental Material files. Please contact the corresponding author directly for reuse.
