# Supplementary figures and images for: Distinct and shared impacts of virulence plasmids on the phenotype and transcriptome in convergent carbapenem-resistant and hypervirulent Klebsiella pneumoniae
Source: Microbiol Spectr. 2026 Jun 9;14(7):e04174-25. doi: 10.1128/spectrum.04174-25 (PMC13340312; doi:10.1128/spectrum.04174-25)

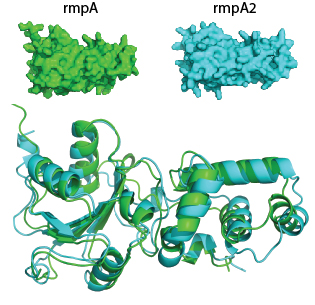

Supplement: Figure S1 — 3D structure and similarity alignment of rmpA and rmpA2 protein. [file spectrum.04174-25-s0001.tif]

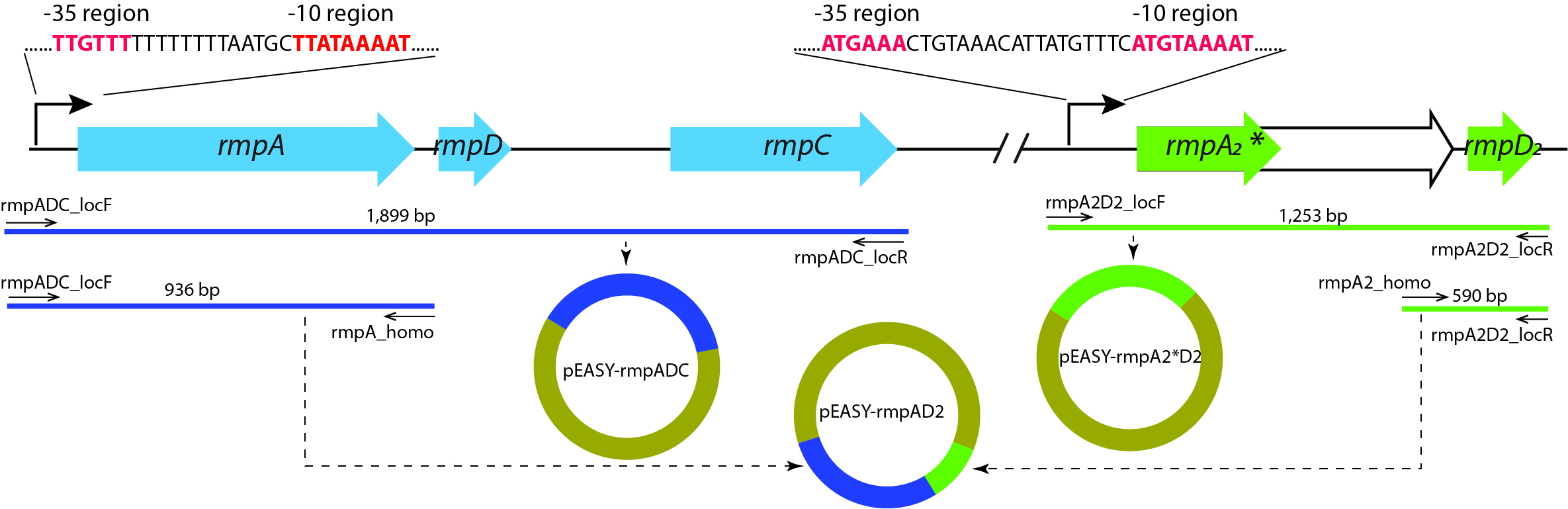

Supplement: Figure S2 — Cloning strategy for rmpADC, rmpA2*D2, and rmpAD2 operons. [file spectrum.04174-25-s0002.tif]
