# Supplementary material for: Distinct and shared impacts of virulence plasmids on the phenotype and transcriptome in convergent carbapenem-resistant and hypervirulent Klebsiella pneumoniae
Source: Microbiol Spectr. 2026 Jun 9;14(7):e04174-25. doi: 10.1128/spectrum.04174-25 (PMC13340312; doi:10.1128/spectrum.04174-25)
Supplement: Supplemental legends — Descriptive legends for Fig. S1 and S2. [file spectrum.04174-25-s0003.docx]

**Fig S1. 3D structure and similarity alignment of rmpA and rmpA2 protein**

Similarity alignment analysis showed rmpA and rmpA2 had close structural similarity with RMSD value of 2.407 Å.

**Fig S2. Cloning strategy for rmpADC, rmpA2*D2 and rmpAD2 operons**

Predicted promoter sequences are shown, with the -35 and -10 consensus elements highlighted in red. Primers and the sizes of the PCR fragments are illustrated.
